# *On metal and ‘spoiled’ wine:* analysing psimythion (synthetic cerussite) pellets (5th–3rd centuries BCE) and hypothesising gas-metal reactions over a fermenting liquid within a Greek pot

**DOI:** 10.1007/s12520-020-01184-1

**Published:** 2020-09-26

**Authors:** E. Photos-Jones, P. Bots, E. Oikonomou, A. Hamilton, C. W. Knapp

**Affiliations:** 1 Archaeology, School of Humanities, University of Glasgow, Glasgow, G12 8QQ UK; 2 Analytical Services for Art and Archaeology (Ltd), Glasgow, G12 8JD UK; 3 Civil and Environmental Engineering, University of Strathclyde, Glasgow, G1 1XQ UK; 4 National Archaeological Museum of Athens, 10682 Athens, Greece

**Keywords:** *Psimythion*, Biotic, Abiotic, Cerussite, Hydrocerussite, Fermenting liquid

## Abstract

A Pb-based synthetic mineral referred to as *psimythion* (pl. *psimythia*) was manufactured in the Greek world at least since the 6th c BCE and routinely by the 4th c BCE. Theophrastus (*On Stones, 56*) describes its preparation from metallic Pb suspended over a fermenting liquid. Psimythion is considered the precursor of one of western art’s most prominent white pigments, i.e. lead white (basic lead carbonate or synthetic hydrocerussite). However, so far, and for that early period, published analyses of psimythia suggest that they consisted primarily of synthetic cerussite. In this paper, we set out to investigate how it was possible to manufacture pure cerussite, to the near exclusion of other phases. We examined the chemical and mineralogical composition (pXRF/XRD) of a small number of psimythion pellets found within ceramic pots (*pyxis*) from Athens and Boeotia (5th–4th c BCE) in the collection of the National Archaeological Museum (NAM), Athens. Analyses showed that the NAM pellets consisted primarily of Pb/cerussite with small amounts of Ca (some samples) and a host of metallic trace elements. We highlight the reference in the Theophrastus text to ‘spoiled wine’ (*oxos*), rather than ‘vinegar’, as has been previously assumed, the former including a strong biotic component. We carried out DNA sequencing of the pellets in an attempt to establish presence of microorganisms (Acetic Acid Bacteria). None was found. Subsequently, and as a working hypothesis, we propose a series of (biotic/abiotic) reactions which were likely to have taken place in the liquid and vapour phases and on the metal surface. The hypothesis aims to demonstrate that CO_2_ would be microbially induced and would increase, as a function of time, resulting in cerussite forming over and above hydrocerussite/other Pb-rich phases. Psimythion has for long been valued as a white pigment. What has perhaps been not adequately appreciated is the depth of empirical understanding from the part of psimythion manufacturers of the reactions between abiotic and biotic components within ‘oxos’/pot, as key drivers of minerals synthesis. Ultimately, psimythion manufacture may rest in understanding the nature of ‘oxos’, antiquity’s relatively little researched strongest acid.

## Introduction

### Psimythion: the sources

There is substantial evidence in the literary record of the Greek world of the 5th–3rd c BCE to suggest that women of all ages and across social boundaries applied a white powder, *psimythion*, as a cosmetic and for purposes of beautification. So extensive was its apparent use that religious temples felt obliged to list it amongst the items that women were not allowed to wear on entering their premises and/or participating in the activities within (Tsoucaris et al. 2011). For example, at the sanctuary of Andania in the Peloponnese (1st c CE), the wearing of psimythion was banned together with gold jewellery and red dye (IG5(1).1390.22).

The desire to have and/or maintain a fair skin appears to have run deeply within Greek culture, as one can gauge from the Homeric poems. The adjective *λευκώλενος* (white-armed) is associated mostly with the goddess Hera (for example Homer *Iliad*, 8.484) but also with mortal women like Andromache, Hector’s wife, or Penelope, Odysseus’ wife, or Helen of Troy and in association with purely female attributes (for example submissiveness, vulnerability, desirability). If a fair skin was indeed a social requisite, for women who did not have it, artificial substances must have been the only remedy.

There is no suggestion that the Homeric ladies used psimythion but it follows that, for later periods, psimythion must have filled that gap in market demand: ‘*Has anyone a dark complexion, white-lead will that correct*,’ Athenaeus (*Deipnosophistai*, xiii.23) notes as late as the 3rd c CE. Before its use as a cosmetic, it is perhaps its ‘whiteness’ that forms the focal point of its attributes as can be gleaned from the earliest reference to psimythion, a fragment of Xenophanes (6th–5th c BCE) (Fragment 28,978a, line 10) and indeed in later texts by Aristotle (*Nicomacheian Ethics* 1096b, line 23).

Nevertheless, the wearing of psimythion came at a price since it could also be a cause of ridicule, particularly by comic poets like Aristophanes, who appears to have had a particular dislike for the substance and/or the women who wore it: *Are you an ape plastered with white lead, or the ghost of some old hag returned from the dark borderlands of death*? (Aristophanes *Eccleciazousae* 1072). It was thought as a means for females to conceal their age: ‘*No, no! as she is there, she can still deceive; but if this white-lead is washed off her wrinkles will come out plainly* (Aristophanes *Plutos* 1065).

Furthermore, it appears that one way to discredit an Athenian lady’s reputation for being virtuous was to allude to her wearing *psimythion*, …. at the wrong time(!): ‘*But it struck me, sirs, that she had powdered her face though her brother had died not thirty days before; even so, however, I made no remark on the fact, but left the house in silence*’ (Lysias, *On the murder of Eratosthenes 1.14).* Men applying psimythion on themselves appears to have been frowned upon: ‘*she adorned his (Alciviades) face like a woman’s with paints and pigments*’ (Plutarch *Alcibiades* 39.2). The use of psimythion by males is also attested by Ctesias, a 5th c BCE Greek physician (Fragment 1b line 689). By the Roman period, the use of *cerussa* (Latin for *psimythion*) seems to have been more widely accepted, yet old beliefs die hard: ‘*You dye your head but you will never disguise your old age nor straighten out the wrinkles in your cheeks. Don’t cover your face with paint so as to have a mask and not a face. For it avails nothing. Why are you so foolish? Paint and dye won’t make Hecuba a Helen*’ (Lucilius *Epigram Book II)*.

It is perhaps its entry to the Hippocratic Corpus of the 5th–4th c BCE that alerts us to its properties beyond the aesthetic. Psimythion was used for external applications. It was dispensed for eye ailments with *spodium*, saffron and myrrh (*Epidemics* 2, 5.22.2); for ear discharge, with *sandarah* and ‘flower of silver’ (*De Morbis* 2, 14.18); for ulcers, mixed with olive oil, resin, pine bark and animal fat (*De Ulceribus* 21.4). Also for gynaecological applications as a suppository, on a piece of wool soaked with water (*De Natura Mulierbi* 29–5). Finally, as an *emplastron* (plaster/wound dressing) and in association with other ‘metallics’ (*misy*, *gold scoria*, *roasted copper* (*De Mulieribus Affectibus* 103, 5–9). Another medical author, Diocles (4th c BCE), includes it in a recipe for eye ailments together with another ‘metallics’ like *pompholyx* (Fragment 147 line 4).

### *Psimythion*: the material culture

Lead- and copper-based minerals can be traced in Egypt in the 2nd millennium BCE (Walter et al. 1999) and in the Royal Tombs of Ur in the 3rd millennium BCE (Hauptmann et al. 2016). The Theophrastus recipe for psimythion making, but also other synthetic minerals, like the copper-based *ios xystos (*Theophrastus, *On Stones, 57)*, puts synthetic mineral manufacture at centre-stage in the Classical/Hellenistic world. Use of psimythion in contemporary art (5th c BCE) is attested only occasionally. It was identified in a single ‘brush stroke’ (‘Pheidias’ brush stroke’) on the pediment of the Parthenon of Athens consisting of hydrocerussite mixed with gypsum and a phosphate mineral (Jenkins and Middleton 1988, 204). More visible are the applications of hydrocerussite in the Hellenistic world, including 4th–3rd c BCE funerary paintings from Macedonia (Brecoulaki et al. 2014) and as an undercoat to organic pigments. At the opposite end of the chronological spectrum, there is evidence for its production, in the Early Bronze Age, at Akrotiri, Thera (Sotiropoulou et al. 2010). Recently, researchers have shown that cerussite had been applied on Early Cycladic marble figurines as a white substrate on the areas of the marble that would be eventually decorated, and prior to the application of the coloured pigment. They suggested that it was the cerussite, rather than the colourful pigments themselves, which may have been responsible for the preservation of anatomical and other details, often described as ‘paint ghosts’ (K Manteli, pers. comm.).

On the other hand, psimythia, in pellet or lump form, have been found within lidded ceramic vessels, in (primarily) female burials. However, such finds tend to be rather rare, when viewed in the context of the vast number of excavated female burials. A number of these pellets have already been analysed, albeit not with the same methods (Table 1). Of the 12 samples, 3 are pink coloured (Eretria, Kerameikos and Delphi) and therefore mixtures: one is cerussite mixed with iron oxide (Eretria), the second, ‘PbO’ (no cerussite/hydrocerussite is mentioned) mixed with HgS (Delphi) and the third, cerussite with HgS ( Kerameikos). Of the remaining 9 samples, 3 are primarily hydrocerussite with small amounts of cerussite (Volos 396 and 471; Agora, Athens). Of the remaining 6, 5 are 100% cerussite (Corinth, Volos 439, Eleusis, Paestum, Kerameikos) and 1 is primarily cerussite with some hydrocerussite (Derveni). Interestingly, the Kerameikos psimythion was found not within a female burial, but rather within that of a male actor; the latter were highly likely to make use of the powder, since males would take on female roles (Kapparis 2018).

**Table:** Table 1

**Site**	**Container Type**	**Date**	**Burial**	**Shape**	**% Cerussite (C) / % Hydrocerussite (H)**	**Reference**
Corinth	Lekanis T3115 with very deep lid.	Late 5^th^ c BCE	Grave 427	Pellets	Cerussite	Shear 1936
Agora-Athens	Vessel OX 219	5^th^–4^rd^ c BCE	Found in grave	Lumps of white pigment	Basic lead carbonate	Caley 1945
Eretria-Euboea	Pyxis CA 508	3^rd^ quarter of the 5th c BCE	Grave of a young girl (?)	Pellets	Cerussite with 10% iron oxide resulting in the pink colour.	Hasselin-Rous et al. 2017
Kerameikos-Athens	Pyxis 10539 or Pyxis 10598	Mid 4^th^ c BCE	Stone sarcophagus (Tomb 24) of the actor Makareus	White pellets	Cerussite	Katsaros et al. 2010
Kerameikos-Athens	Pyxis 10537	Mid 4^th^ c BCE	Stone sarcophagus (Tomb 24) of the actor Makareus	Pink pellets	Cerussite:84%. Cinnabar: 14%	Bimbenet-Privat et al 2009
Volos 396 Demetrias	Pyxis BE 155444	4^th^–3^rd^ c BCE	Female pit grave 396.	Lumps of white compact powder	C/H= 18/82	Welcomme et al. 2006
Volos 471 Demetrias	Pyxis BE15545	4^th^–3^rd^ c BCE	Female pit grave 471	Lumps of white compact powder	C/H=16/84	Welcomme et al. 2006
Volos 439 Demetrias	Lekanis BE 22624	4^th^–3^rd^ c BCE	Female burial in tile grave 439.	Lumps of white compact powder	Cerussite :100%.	Welcomme et al. 2006
Eleusis	Lekanis	Greco–Roman	Female burial within sarcophagus	Two small discs in small box	Cerussite :100%.	Welcomme et al. 2006
Derveni. Macedonia	E32A deposit of an intense white powder	4th quarter of the 4th c BCE	Female cist tomb B	White powder	C/H= 96/4	Welcomme et al. 2006
Paestum, Italy	Lekanis T4/1990	Greco–Roman	Female tomb 4	White powder	Cerussite : 100%.	Welcomme et al. 2006
Delphi	Pyxis	400–350 BCE	Burial of the theatre actress	Pink powder	‘PbO’ with cinnabar	Liritzis et al. 2018

The analyses of the artefacts in Table 1, by different researchers and over the last 80 years, suggest that 6 out of the 9 white samples contain cerussite over and above hydrocerussite and/or other phases. The above data set is not large and, as such, conclusions drawn are more likely to be indicative, rather than definitive. Nevertheless, the question arises as to which parameter(s) control the production of cerussite, above other phases, within the Theophrastus pot. It is the excess of CO_2_ and maintenance thereof throughout the 10-day cycle that would have ensured the production of cerussite in preference to the hydrocerussite. This paper sets out to present a working hypothesis regarding the importance of the biotic component within the *oxos* as a driver of CO_2_ production. The two sections that follow assess past reconstructions of the Theophrastus pot (‘*Oxos* in the Theophrastus recipe: Pb metal in the presence of fermenting liquid’ section) and recent (‘Vinegar in Pb corrosion studies’ section) work on Pb metal corrosion led by acetic acid bacteria (AAB) and other microorganisms.

### *Oxos* in the Theophrastus recipe: Pb metal in the presence of fermenting liquid

Theophrastus’ (*On Stones*, 56) recipe for psimythion making reads as follows: ‘lead (*molybdos*) about the size of a brick is placed in jars (*pithos*) over vinegar (*oxos*) and when this acquires a thick mass, which it generally does in ten days, then the jars are opened and a kind of mold (*evros)* is scraped off the lead, and this is done again until it is all used up. The part that is scraped off is ground in a mortar and decanted frequently, and what is finally left at the bottom is white lead (*psimythion*)*’* (Caley and Richards 1956, 188) (the transliterated Greek in parentheses is our addition.)

There have been many attempts to reproduce psimythion experimentally and in the manner of the Theophrastus set-up. Katsaros et al. (2010) reproduced this recipe in a similar pot, a pithos, resulting in the production of hydrocerussite and cerussite. When the authors analysed psimythion pellets from the Kerameikos cemetery (see Table 1), they found them to consist primarily of cerussite.

Principe (2018) carried out experiments similar to that of Katsaros et al. (2010) but in a glass jar. He did not report any mineralogical analysis of his finds, so it is not clear what exactly he made, cerussite or hydrocerussite or lead acetate. Regarding the question of the source of the CO_2_, he suggests that the diurnal effect, i.e. the temperature variations between day and night, would have had an effect on the O_2_/CO_2_ introduced into the poorly sealed vessel of coarse fabric every night (‘a daily cycle of breathing’) as a result of vinegar vapour contraction. CO_2_ may indeed have been made available in that way but the question is whether it would be sufficient to generate enough hydrocerussite/cerussite over a 10-day cycle. We argue in favour of a more reliable and continuous source of CO_2_, i.e. that which can be generated within a fermenting liquid by either aerobic (acetic acid) bacteria converting acetic acid to CO_2_ and water and/or facultative anaerobic yeasts (*Saccharomyces*) doing the same.

We have introduced the presence of the microorganisms because Theophrastus makes reference to the use of ‘*oxos*’. Within a 5th–3rd c BCE context, *oxos* would have been translated as ‘poor wine or vin ordinaire’ or ‘vinegar produced from oxos’ (see entry in Liddell Scott Dictionary—www.perseus.tufts.edu). In the second meaning, it is made clear that vinegar is distinct from and not synonymous with *oxos.* Xenophon in his *Anabasis* (2.3.14) refers to *oxos* produced from ‘boiling’ (fermenting) palm wine. The issue of fermentation, both implied and stated by these authors, suggests that the biotic component plays a key role and needs to be brought forward in any psimythion-related discussion.

In the past, a number of researchers had queried whether the Theophrastus arrangement could actually produce anything other than lead acetate, the result of acetic acid vapour reacting with lead metal (Bailey 1932; Shear 1936; Stevenson 1955). Since lead acetate, as a water-soluble salt, would have never worked as a cosmetic or an artist’s pigment, it follows that there must have been an obvious source of CO_2_ helping to convert lead acetate to lead carbonate.

In an attempt to tackle the question of the origin of the elusive CO_2_ source, Caley and Richards (1956, 188) proposed that ‘another source of the carbon dioxide may have been the so-called vinegar used in the process. If this was merely a spoiled grape juice undergoing both alcoholic and acetous fermentation, ample carbon dioxide would have been available’.

Grape skin hosts a number of aerobic (acetic acid bacteria (AAB)), facultative anaerobic (e.g. yeasts, like *Saccharomyces* spp.) and anaerobic (e.g. *Acetobacterium* spp.) microorganisms which play key roles in the conversion of sugars to alcohol and alcohol to vinegar. Mortimer and Polsinelli (1999) have demonstrated that one of them, *Saccharomyces cerevisiae*, constitutes about one-quarter of the totality of yeasts living on damaged grape skin (grapes that have been pressed and squeezed)*.* Cavalieri et al. (2002) identified the same microorganism in the lees residue within a wine jar dated to the 4th millennium BCE from Egypt. The authors have suggested that this yeast served as an inoculum for bread and beer. Although yeasts are responsible for the transformation of grape juice to wine they are also capable of spoiling it. *S. cerevisiae* is the yeast primarily responsible for spoilage since it can resist high alcohol concentrations and low pH (Martorell et al. 2005).

### Vinegar in Pb corrosion studies

In their study on the crystal growth of lead carbonates under different media, Sánchez-Navas et al. (2013) reproduced the ‘stack’ or ‘Dutch’ process where metallic lead was first oxidized by acetic acid vapours in the presence of moisture, and the resulting lead acetate was later transformed into basic lead carbonate (hydrocerussite) by the action of carbon dioxide (Gettens et al. 1967). The ‘stack’ process used the same raw materials, i.e. lead metal exposed to acetic acid vapours, but not in a closed pot, as in the Theophrastus case. The source of the CO_2_, in this case, was manure which surrounded the stacked pots of lead/acetic acid.

Sánchez-Navas et al. (2013) used various sources of CO_2_, one of them being a liquid culture of *Acetobacter* sp., or acetic acid bacteria (AABs) which can make acetic acid from alcohol but can also oxidize acetic acid for the production of CO_2_ and H_2_O. They considered this a bio-mediated process and noted that if nitrogenated organic matter is present (for example proteins), then not only CO_2_ but also NH_3_ is produced. Ammonia (in the gas phase) would increase the pH on the metal plate favouring, they argued, the formation of hydrocerussite. They also noted that, in the course of their reactions, the product formed was poorly crystalline hydrocerussite/cerussite at atmospheric partial pressures of CO_2_ (10^–3.5^ atm) but when a higher CO_2_ pressure was used (1 atm) by flowing gas in the container, cerrusite was formed. This is consistent with the higher carbonate content of cerussite, where 1 mol of Pb requires 1 mol CO_2_, compared with 0.67 mol of CO_2_ required per mole of Pb to form hydrocerussite.

More recently, Gonzáles et al. (2019b) also reported that a CO_2_ producer is necessary to quickly form lead carbonates. They showed that the metal  surface can have many layers of product consisting of plumbonacrite (Pb_5_(CO_3_)_3_O(OH)_2_) at the lead surface followed by outer layers of hydrocerussite Pb_3_(CO_3_)_2_(OH)_2_ and cerussite (PbCO_3_) which are in direct contact with each other. The role of CO_2_, heat and UV light in the production of cerussite deserves further study. They noted that using vinegar as the source of acetic acid, and with no separate CO_2_ source beyond atmospheric CO_2_, no hydrocerussite or cerrusite is produced, even after 1 month; yet Katsaros et al. (2010), by placing lead over vinegar in a jar sealed but with a breathable leather lid, formed hydrocerussite and cerussite after 10 days. Both systems had access to atmospheric CO_2_ and O_2_, the main difference being that Katsaros et al. heated their sample container (27–55 °C) by placing it outside ‘in the sun’, followed by washing and drying the sample outside in sunlight. Washing removes soluble acetates which do not convert on heating to carbonates (Martínez-Casado et al. 2016) but whether heating/sunlight converts plumbonacrite to cerussite is unknown. Finally, Gonzáles et al. (2019b)) addressed the question of hydrocerussite stability in air and reported no evidence at all of cerrusite formation after hydrocerrusite was exposed to laboratory air for 26 months; it is therefore unlikely that the cerussite analysed here was formed during burial.

**Fig. 1:**
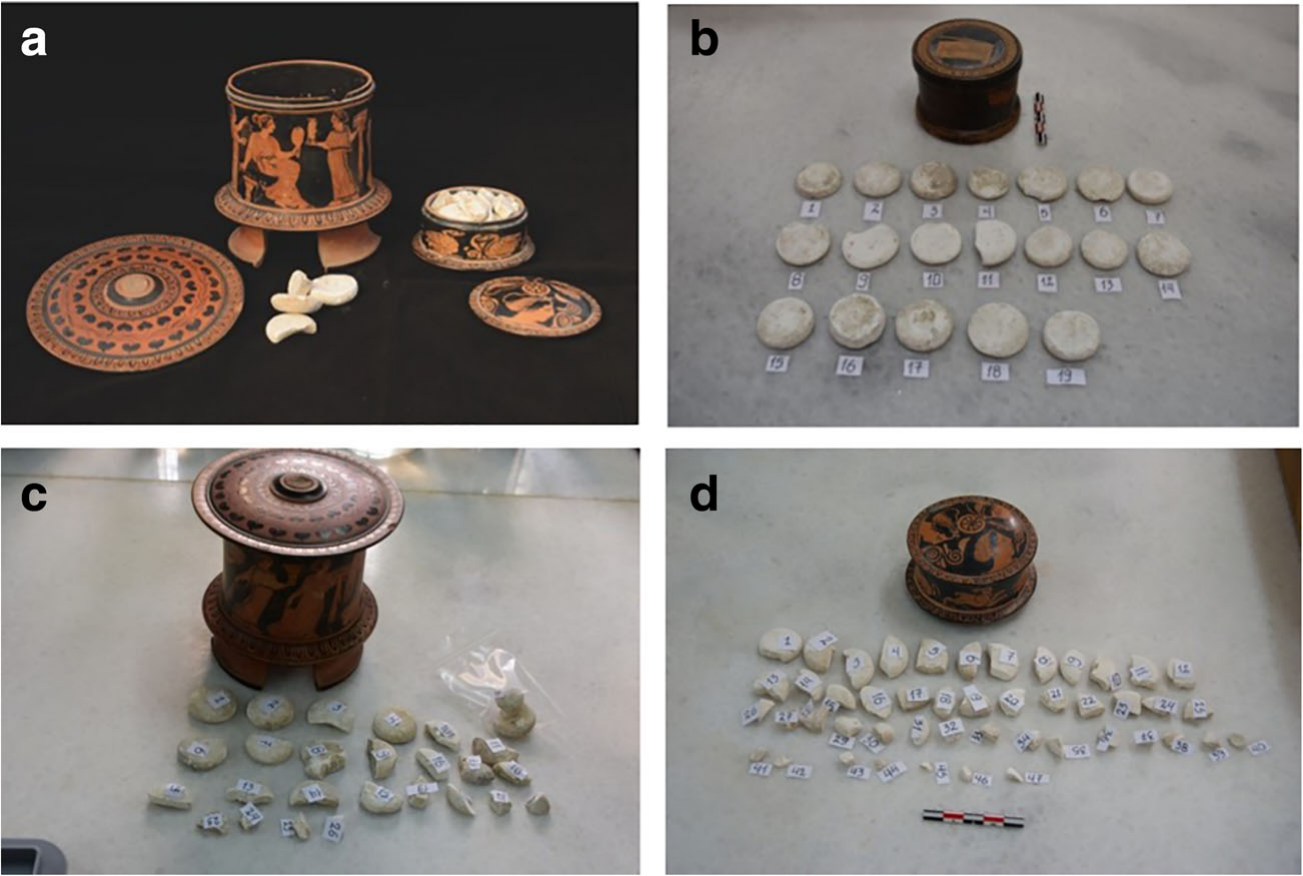
**a** Pyxides NAM 13676a and 13676b (Photograph, courtesy of National Archaeological Museum, Athens). **b** Pyxis NAM 13676a with associated complete, near complete and fragments of psimythion pellets numbered 1–26. **c** Pyxis NAM 13676b with associated complete, near complete and fragments of psimythion pellets numbered 1–47. **d** Complete, near complete and fragments of psimythion from Tanagra Boeotia, numbered 1–19. They are not associated with any particular pyxis

**Fig. 2:**
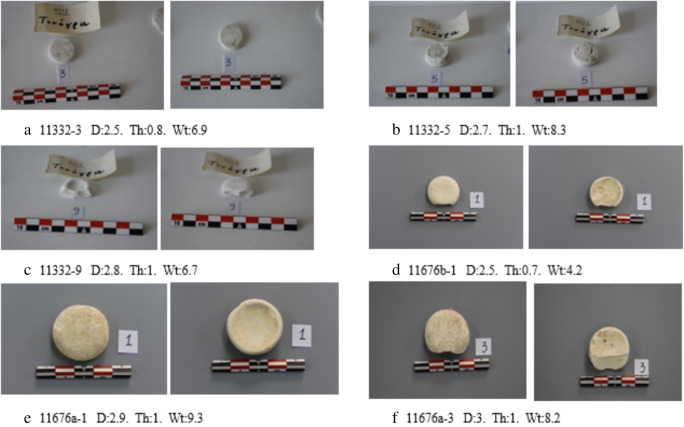
Dimensions (cm) and weight (g) of a number of complete psimythia showing different sections and presumably ‘cast’ in variously shaped moulds. See also Fig. 3

**Fig. 3:**
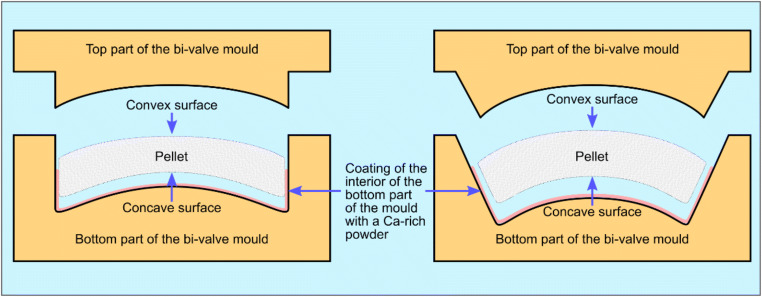
Suggested reconstruction of the bivalve mould used for the production of psimythia. Coating of the interior of the mould for easier pellet removal is also suggested, but may have not been applicable in all cases

**Fig. 4:**
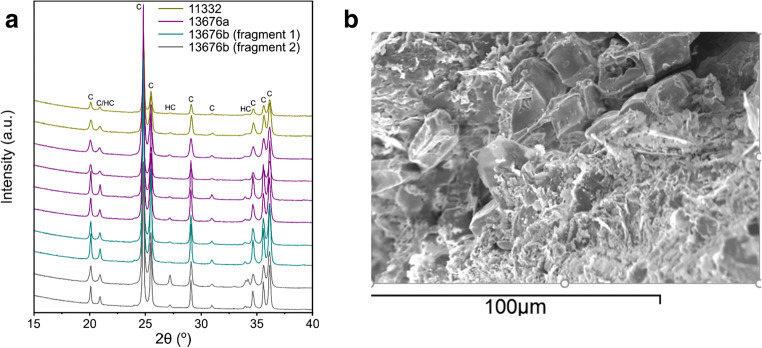
**a** XRD patterns of pellets from 11332, 13676a and 13676b. **b** SEM-EDAX image and analysis of the surface of 11332b showing large well-formed cerussite crystals

This paper is divided in two parts. In the first part, we investigate a select number of complete and fragmented pellets of psimythion, in the collection of NAM (‘The NAM pellets’ section and Fig. 1), on the basis of their composition, as well as metrology (‘The NAM pellet metrology’ section, Fig. 2, Table 2). The pellets appear to have been ‘cast’ in moulds and therefore it is possible to arrive at the shape of the latter by looking closely at the shape of the former (Fig. 3). The ‘Method’ section outlines the method of their examination while the ‘Results’ section gives a description of the results. Chemically the pellets are made of Pb with Ca as a minor element (‘pXRF analyses of multiple fragments’ section and Tables 3 and 4). Mineralogically they are made primarily of cerussite (‘XRD and SEM-EDAX analysis’ section and Table 5 and Fig. 4a). In hypothesising a mechanism by which cerussite could be produced to the near exclusion of other phases, we focus on the biotic element within the ‘*oxos’*, as a key driver in CO_2_ production within the closed Theophrastus pot. For a full discussion, see the ‘CO_2_ -rich conditions prevailing within the Theophrastus pot: a hypothesis’ section.

**Table 2:** List of dimensions and weights for 12 complete psimythion pellets from Tanagra (11332) and 3 complete pellets from Athens pyxis 13676a

NAMA no.	Diameter (cm)	Thickness (cm)	Weight (g)
11332-1	2.8	0.8	7.7
11332-2	2.5	0.7	6.1
11332-3	2.5	0.8	6.9
11332-4	3	0.7	6.7
11332-5	2.7	1	8.3
11332-6	2.8	0.8	6.6
11332-7	2.9	0.7	6.3
11332-12	2.9	0.6	5.8
11332-14	2.8	0.6	5.9
11332-19	2.8	0.7	7.2
11332-20	2.7	0.7	6.8
11332-21	2.8	0.6	5.4
13676a-1	2.9	1	9.3
13676a-2	2.9	1	8.4
13676a-4	2.8	0.9	7.8
Mean	2.79	0.77	7.01
s.d.	0.15	0.14	0.15

**Table 3:** Calibrated pXRF values for Pb and Ca in weight % of 9 pellets from 13676b and 9 from 11332. Both faces are analysed separately: face A = flat (convex) face; face B = curved (concave) face. The purpose is to highlight possible variations in these two elements between the two faces. The results show higher Ca concentrations in the 13656b samples but with no marked differences between faces. No calcium rich phases were detected with the XRD (see below)

Sample face A = flat; face B = curved	Calibrated values for Pb	Calibrated values for Ca	Sample face A = flat; face B = curved	Calibrated values for Pb	Calibrated values for Ca
11332-11 face A	88.31	0.21	13676b-1 face A	65.25	6.80
11332-10 face A	79.50	0.66	13676b-2 face A	87.13	0.42
11332-09 face A	81.47	0.36	13676b-3 face A	73.61	3.97
11332-08 face A	90.87	0.59	13676b-4 face A	89.10	0.49
11332-07 face A	94.24	0.17	13676b-5 face A	86.58	1.56
11332-06 face A	98.48	0.15	13676b-7 face A	86.16	1.05
11332-05 face A	88.81	0.87	13676b-8 face A	83.76	0.93
11332-04 face A	85.34	0.59	13676b-9 face A	87.60	0.45
11332-03 face A	86.75	0.25	13676b-10 face A	81.30	0.33
Mean	88.20	0.43	Mean	82.28	1.78
11332-11 face B	87.41	0.25	13646b-1 face B	83.58	5.02
11332-10 face B	89.74	1.09	13646b-2 face B	76.15	0.75
11332-09 face B	86.74	0.95	13646b-4 face B	61.37	2.33
11332-08 face B	85.59	0.65	13646b-5 face B	64.61	3.81
11332-07 face B	85.50	0.75	13646b-7 face B	64.31	3.31
11332-06 face B	82.11	0.27	13646b-8 face B	58.28	1.26
11332-05 face B	80.94	0.43	13646b-9 face B	75.70	0.32
11332-04 face B	83.16	1.00	13646b-10 face B	76.33	2.73
11332-03 face B	94.57	0.44	Mean	70.04	2.44
Mean	86.20	0.65

**Table 4:** Uncalibrated pXRF values for trace elements in ppm in pellets from 13676b and 11332

Sample	Cu	Sb	Sn	Cd	Ag	Sample	Cu	Sb	Sn	Cd	Ag
13676b-1	281	4007	4085	2344	1702	11332-3	357	5406	4868	3204	2156
13676b-2	250	4005	3578	2058	1742	11332-4	282	4909	4445	2613	1921
13676b-3	260	3325	3436	1858	1884	11332-5	277	4923	4675	3094	1908
13676b-4	371	3562	3392	1918	1602	11332-6	231	5349	4995	3466	2348
13676b-5	267	3996	3933	2389	1561	11332-7	315	5506	4542	3238	2009
13676b-6	330	3011	3335	1687	1479	11332-8	318	5154	4646	3120	2205
13676b-7	451	3561	3372	1893	1562	11332-9	318	4908	4518	2804	1973
13676b-8	358	3363	3349	1901	1607	11332-10	371	4925	4463	2908	1885
13676b-9	270	4129	4058	2453	1618	11332-11	217	5377	4568	2896	2021
13676b-10	464	3234	3366	1772	1499						
Mean	330	3619	3590	2027	1626	Mean	298	5162	4636	3038	2047
S.d.	79	391	310	273	121	S.d.	52	250	187	258	157
% s.d.	23.97	10.81	8.64	13.45	7.47	% s.d.	14.57	4.63	3.83	8.05	7.27

**Table 5:** Powder XRD data and Rietveld refinement for a selection of psimythion fragments

Sample	Weight % cerussite	Weight % hydrocerussite
13676a—powder sample scraped off from convex face	99.3 ± 0.1	0.7 ± 0.1
13676a—powder sample scraped off from concave face	98.1 ± 0.1	1.9 ± 0.1
13676b—powder sample scraped off from convex face—fragment 1	99.7 ± 0.1	0.3 ± 0.1
13676b—powder sample scraped off from concave face—fragment 1	98.7 ± 0.2	1.3 ± 0.2
13676b—powder sample scraped off from convex face—fragment 2	97.0 ± 0.2	3.0 ± 0.2
13676b—powder sample scraped off from concave face—fragment 2	88.9 ± 0.2	11.1 ± 0.2
13676a—powder sample 1	97.6 ± 0.2	2.4 ± 0.2
13676a—powder sample 2	98.1 ± 0.2	1.9 ± 0.2
11332—powder sample 1	97.8 ± 0.3	2.2 ± 0.3
11332—powder sample 2	97.9 ± 0.2	2.1 ± 0.2

### The NAM pellets

Complete and fragmented pellets of psimythion, deriving from the contents of two pyxides, lidded ceramic vessels, *NAM 13676a* and *NAM 13676b* (Fig. 1a) were retrieved from burials dating to the 5th–4th c BCE, Athens; also ‘loose’ (without a pyxis) complete and fragmented pieces of psimythion (Fig. 1d) from a cemetery at Tanagra, Boeotia, c. 60 km north of Athens. The pyxis (NAM 11447), with which the latter are currently displayed, is from Rhodes. These pyxis-less pellets from Tanagra are also dated to the 5th–4th c BCE.

#### NAM 13676a (Fig. 1b)

Pyxis with lid found together with pyxis B, 136763b, in the foundations of a house in a plot opposite the National Technical University, Patission Street, Athens, in the late 1890s. On the body of the pyxis (Fig. 1b) is a depiction of women’s quarters, while on the lid, a band of egg-and-dot patterns surrounds a wreath of ivy leaves. The pyxis is attributed to the Painter of Athens 1585 and is dated 410–400 BCE. The 13676a collection consists of three complete pieces, one near-complete and 22 fragmented ones, a total of 26.

#### NAM 13676b (Fig. 1c)

Miniature pyxis with lid found together with pyxis A, 136763a above. On the body is a depiction of a hare, a feline and swans, while on the lid, there are three female heads alternating with anthemia surrounded by a band of egg-and-dot pattern. It is also dated 410–400 BCE. Its contents include two complete pieces, two near complete and numerous fragmentary ones.

#### NAM 11332 (Fig. 1d)

Thirty-six white pellets from Tanagra, Boeotia: 14 of them are complete, 14 are near complete and eight are fragmentary. Figure 1d displays 19 of the 36. They are dated, by association, to the end of the 5th–end of 4th c BCE based on the chronology of the tombs in the Tanagra cemetery.

## Method

### pXRF

pXRF analyses took place at the National Archaeological Museum, Athens, with a NitonX3Lt-GOLDD instrument which has a 50kVAgX-raytube, 80-MHz real-time digital signal processing and two processors for computation and data storage respectively. The TestAllGeo (TAG) mode was selected. Analysis time was set at 60 s and two measurements were taken on different spots on each fragment. An average of the two is shown here. Replicate analyses of NIST SRM 2709a soil standard revealed satisfactory precision (< 10% Zr and < 5% Rb and Sr), good accuracy for Sr (< 10%), but poor accuracy for Zr and Rb which were underestimated by over 90%, based on using NIST 2709a as the internal standard. However, the above elements are not crucial to the discussion here. The two elements of relevance here were Pb and Ca. Therefore, four external standards were prepared using reagent grade PbO and CaCO_3_. Results are shown in Table 3.

### SEM-EDAX

A tungsten filament Scanning Electron Microscope (W-SEM) HITACHI S-3700, combined with an Oxford Inca 350 with 80-mm X-Max detector, based at University of Strathclyde’s Advanced Materials Research Laboratory, was used for the elemental analysis of materials. Freshly fractured surfaces of specimens were gold -coated.

### XRD

Fragments of psimythion pellets were analysed using X-ray diffraction (Bruker D8 Advance) with a Cu K_α_ X-ray source. To determine the cerussite to hydrocerussite ratios within the analysed replicates, we performed quantitative Rietveld refinements on the XRD patterns using TOPAS and the crystallographic information files for cerussite (Antao and Hassan 2009) and hydrocerussite (Martinetto et al. 2002).The subsamples from NAM 11332, NAM 13676a and NAM 13676b consisted respectively of powdered psimythion only, both powdered and a single fragment of a psimythion pellet and two fragments of one (or two) psimythion pellet(s).

### DNA Analyses

Given the presence and crucial role that microorganisms play in the reactions to be outlined below, it was desirable to find potential DNA signatures of the microorganisms within the samples. DNA half-life has been determined to be over 500 years (Allentoft et al. 2012), but, buried in soils, it can last 1000–10,000 years (Thomsen and Willerslev 2015). DNA were extracted from approximately 100 μg of mineral material in triplicate using a Qiagen DNAeasy Soil extraction kit. DNA quantity and extraction purity were screened using UV-microspectrometry (Epoch BioTek; Swindon, UK). Final extracts were further diluted 1/10 and 1/100 to be included in downstream processes along with the ‘neat’ (undiluted) extracts. Dilution of samples has often been applied in cases where enzyme-inhibiting materials (e.g. chlorophyll, metals or humic acids) could possibly exist. Here, the likelihood of Pb^+2^ posed a concern, although the extraction method does involve the precipitation removal of cationic elements.

Assays for the detection of *Saccharomyces* fungus and *Acetobacter* involved sensitive quantitative polymerase chain reactions (qPCR). Primers were based on those previously reported: *Saccharomyces* sp. (SFC1 forward primer: 5′ GGACTCTGGACATGCAAGAT and SCR1 reverse primer: 5′ ATACCCTTCTTAACACCTGGC; Salinas et al. 2009) and two sets of *Acetobacter* sp. primers: (forward #1: GCTGGCGGCATGCTTAACACAT and reverse #1: GGAGGTGATCCAGCCGCAGGT; forward #2: TCAAGTCCTCATGGCCCTTATG and reverse #2: TACACACGTGCTACAATGGCG (González et al. 2005). qPCR conditions involved 10-μL reactions (5 μL GoTaq® qPCR Master Mix; Promega (Madison, WI, USA)) with 40 cycles of thermal cycling: 95 °C for 10 s (DNA denaturation), 60 °C for 20 s (primer annealing and elongation), on a BioRad iCycler (BioRad; Hercules, CA, USA). Genomic DNA from previously identified cultures were used as positive controls; molecular-grade water was used as a negative control.

## Results

### The NAM pellet metrology

Twelve pellets from Tanagra, Beoetia (11332) and three pellets from Athens (13676a) showed surprising homogeneity with a mean diameter of 2.8 cm, thickness of 0.8 cm and a weight of 7 g and with a uniform standard deviation of c. 15%. Interestingly, our data is in good agreement with those of Katsaros et al. (2010) who measured pellets from the Kerameikos. They report diameter = 2.75 cm, thickness = 0.6 cm and weight = 5.5 g suggesting that the psimythion industry of the period operated on an accepted ‘standard’ of weights and measures (Table 2).

The shape of the pellets varies and as long as the dimensions and weights are kept constant, it is possible to make allowances for ‘preferred’ shapes by different workshops. The Tanagra pieces vary as follows: 11332-3 (Fig. 2a) is flat on both sides, while Fig. 2b and c show pellets with only one side concave and the other flat. The Athens pellets had a shallow convex/concave surface, with the concave surface facing up (Fig. 2e—right) and the convex surface in the opposite direction (Fig. 2e—left); similarly for those in Fig. 2d and f. The different cross-sections displayed by the pellets suggest that each workshop may have had its own preferred shape(s), but all were required to abide by prescribed dimensions and weight.

The uniform shape and size of the NAM pellets suggest that they may have been formed within a bivalve mould, as shown in Fig. 3, the top part closing on the bottom in the manner shown here: the concave surface facing down and adhering onto the bottom section of the mould, and the convex adhering to the top part. The mould may have been made of carved wood or ceramic given the smooth surface of all pellets. The mould may have been covered by a medium, perhaps calcium-rich, preventing the adherence of the pellet on the mould and allowing each pellet to detach easily. The space left by an air bubble trapped between the surface of psimythion and the bottom section of the mould can be seen in Fig. 2d.

### pXRF analyses of multiple fragments

Non-destructive pXRF analysis was carried out on a number of samples, at the NAM, both complete and fragmented (Tables 3 and 4). In total, c. 9 pellets of sample 13676b and 9 pellets of sample 11332 were analysed on both their flat and curved faces. The results were calibrated against prepared standards (PbO-CaCO_3_) for only two elements Pb and Ca (Table 3) which showed values above 0.5%. The 13676b samples contained c. 82% Pb and 2% Ca by weight on their flat face; and c. 70% Pb and 2.5% Ca on their curved faces. Ca values for the 11332 samples were low and there was no variation between the curved and the flat face. Table 4 shows uncalibrated data sets for same sample sets and with regard to Cu, Sn, Sb, Cd and Ag.

### XRD and SEM-EDAX analysis

Permit was granted to take one fragmented pellet from each of the three pyxides 13676a and 13676b and 11332 and subject it to destructive analysis via SEM-EDAX and XRD.

SEM-EDAX image and analysis (Fig. 4b) of the surface of a fragment of a pellet from 11332b showed large well-formed cerussite crystals with composition corresponding to c. 70% Pb, 15% O and 15% C; the results are uncalibrated but definitely point to cerussite. For XRD analysis, a number of samples were obtained. In the case of the pellet from pyxis, 13676a, powder samples were taken from both surfaces, the convex and concave. A section of that pellet was subsequently crushed and ground and mounted for XRD analysis. Similarly for pellets from pyxides 13676b and 11332. The XRD patterns for all are shown in Fig. 4a and the quantitative assessment in weight per cent of each is given in Table 5. Only two minerals are identified, namely cerussite and hydrocerussite. It is noted that the powder scraped off the concave surface has a slightly higher concentration of hydrocerussite as opposed to powder scraped off the convex surface. In one case, the hydrocessurite of that concave face is c. 11%. When the powder subsample is obtained following pulverisation of the original, then the hydrocerussite concentration is c. 2%.

### Summary

The entire collection of NAM *psimythia* consists of 103 pieces, in complete or mostly fragmentary state. XRD analysis of three pellets (various fractions) shows cerussite with hydrocerussite not exceeding c. 11%. Ca has been detected in the pXRF but was not visible in the XRD of the samples analysed here and as part of a distinct phase. From the perspective of trace elements, there are elevated amounts of Ag which may point to an Ag-rich Pb metal source, Laurion in Attica being the most likely candidate for the manufacture of such metal (Photos-Jones and Jones 1994).

### DNA extraction and qPCR of some pellets

Community DNA was extracted from three pellets. Visually, the material had minimal evidence of organic matter, and determinations of DNA concentrations were found below detection (< 2 ng/μL) and without any evidence of impurities. The lack of DNA evidence does not suffice to preclude the qPCR analysis, as the assay is innately more sensitive (detection limit: 100 DNA copies/g). However, the samples were negative (positive assays showed results > 95% efficiency of reactions). As such, *Saccharomyces* or *Acetobacter* were absent in all pellets. This suggests that reactions on the metal-mineral phase are likely abiotic, but it may be that the effect on the composition of gases within the pot may have been largely microbially mediated.

## CO_2_-rich conditions prevailing within the Theophrastus pot: a hypothesis

Theophrastus describes psimythion manufacture as consisting of two stages: (a) the preparation of synthetic cerussite and (b) at the beneficiation thereof by grinding, dissolving and decanting of the soluble components with the aim of their enrichment and refinement with regard to both composition and particle size. The need to grind the flakes of psimythion into fine powder, its subsequent mixing with water allowing for any soluble matter to dissolve, the settling of the insoluble parts and the decanting of the soluble ones, all of the above steps would have aimed at producing a pure final product.

**Fig. 5:**
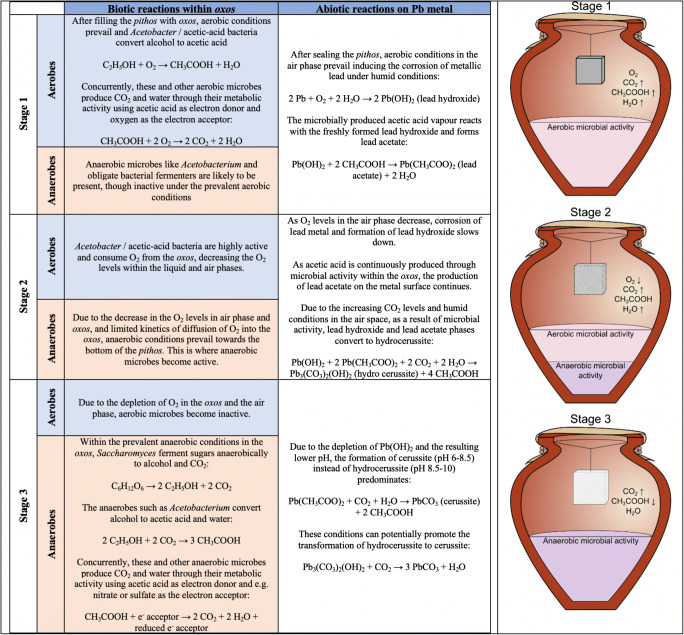
Reactions, biotic and abiotic, taking place on the metal surface as a function of time in a 10-day cycle

Our proposed model for the reactions taking place within the Theophrastus pot is illustrated in Fig. 5. The prescribed 10-day cycle has been divided notionally into three stages to account for reactions taking place at different times and ‘fronts’. These stages are not sharply divided but rather merge into one another and can also be reversible, if conditions within the pot change. First, there are reactions between the metal (lead) surface and the gaseous phase (i.e. the air space above the liquid). Second, there are reactions taking place within the *oxos*. The biotic component is made up of microorganisms, both aerobic (acetic acid bacteria (AAB)/*Acetobacter*) and anaerobic (yeast/*Acetobacterium* and other obligate bacterial fermenters) actively changing the chemistry of the *oxos*. These changes result in changes in the gas phase, via the production of O_2_/CO_2_/acetic acid vapour, which in turn have a direct effect on the reactions on the metal surface.

In **stage 1**, aerobes (AAB and *Acetobacter*) are active in an oxygen-rich environment converting alcohol to acetic acid. But the same bacteria also respire aerobically converting acetic acid to CO_2_ and water. Although at the start the gas phase in the pot is O_2_ rich, under the dual action of the aerobes (oxidation of alcohol and respiration/metabolic activity), it becomes increasingly richer in CO_2_, acetic acid and water vapour. On the metal surface, a reaction between Pb (metal), O_2_ and water vapour results in the formation of lead hydroxide; this, in turn reacts with a vapour that is an increasingly rich in acetic acid, forming lead acetate.

Stages 1 and 2 form a continuum, in the sense that there is no abrupt end to stage 1 before stage 2 begins. The reactions on the Pb metal surface continue to produce lead hydroxide (or lead oxide) and acetate; given the increased levels of CO_2_ (due to the microbial respiration/metabolic activity), hydrocerussite and acetic acid are formed during stage 2. At the end of stage 1, O_2_ levels have depleted due to microbial consumption and the formation of lead hydroxide stops. During stage 2, anaerobic microbes are (re)activated in the bottom of the *pithos* and due to the depletion of O_2_, they will be active throughout the *pithos* during stage 3. In stage 3, the reactivation of the anaerobes within *oxos* leads to the formation of additional levels of CO_2_ due to the anaerobic respiration/metabolic activity by microbes converting acetic acid into CO_2_ and H_2_O. During the depletion of lead hydroxide (through the formation of hydrocerussite) and the increase in the levels of CO_2_, cerussite is formed at the expense of hydrocerussite. We should note, however, that we cannot rule out or confirm the formation of hydrocerussite via plumbonacrite, as an intermediate phase, as observed by Gonzáles et al. (2019a) during the Dutch process.

**Fig. 6:**
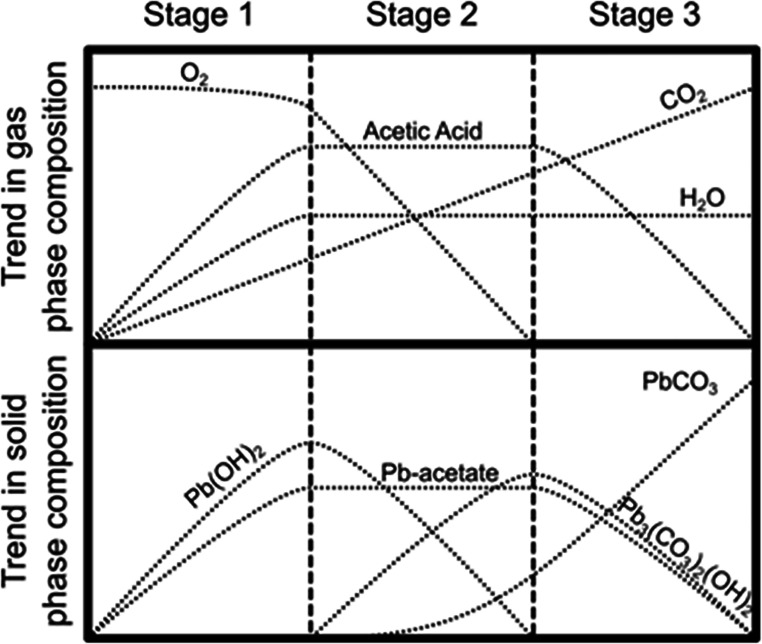
Trends in gas and solid phase composition as a function of 10-day cycle of events within the pot for making psimythion according to Theophrastus

Figure 6 gives an illustrative summary of the trend in gas and solid phase composition for stages 1–3 as described in Fig. 5. Stage 1 is characterized by a slow decrease in O_2_, rapid increase in acetic acid vapour and gradual increase in CO_2_. Lead hydroxide and lead acetate form on the lead metal surface. During stage 2, there is a levelling in the amount of acetic acid vapour, together with a sharp decrease in O_2_ and a continuing increase in CO_2_. On the metal surface, lead hydroxide gets depleted as hydrocerussite forms rapidly followed by an initially slow rise in lead carbonate. Finally, in stage 3, acetic acid vapour is gradually depleted, while CO_2_ levels continue to rise leading to the preferential formation of lead carbonate (cerussite), rather than basic lead carbonate (hydrocerussite) on the ‘corroded’ lead metal surface.

## Conclusions

The manufacture of a white lead-based pigment has had a long history, recorded in detail, at least since the 4th c BCE. Given the commonality of its raw materials, i.e. lead metal and *oxos*, the simplicity of the installations and the relatively hands-off nature of the process, there has been a broad, albeit expressly unstated, assumption that from antiquity to the modern era ‘one recipe’ fitted all. This is not true and earlier researchers took pains to recognize and report on the many variations within that long time-span, not simply chronologically, but also regionally (Pulsifer 1888, 196). We suggest that during that time-depth ‘different’ white lead-based minerals were produced and each period may have developed its own recipes and working conditions. Mass production of this white pigment continued well into the modern times via the stack/Dutch process (Gettens et al. 1967). We argue that present archaeological evidence suggests that, for the period of concern here, synthetic cerussite was the main mineral intended to be produced. The question is how was this achieved.

The proposed hypothesis for the conditions prevailing within the Theophrastus pot is that they are dynamic and not static throughout the 10-day cycle. Active (and inactive) microbial communities within the *oxos* control the composition of the gas phase and in turn are controlled by it. This dynamic state must have been well understood by the psimythion makers. Any disturbance thereof, even a mere opening of the lid at any stage in the 10-day cycle to ‘check progress’, or indeed any interruption of the process somewhere between stages 2 and 3 (with subsequent introduction of O_2_) would alter the dynamics and probably push towards the production of the hydrocerussite, at the expense of cerussite. The above consistent ‘push’ of the equilibrium towards cerussite combined with the standardisation of the pellet form, shape and weight (see the ‘The NAM pellet metrology’ section) suggests an industry well on top of its own practice.

Returning to the NAM artefacts and our search for *saccharomyces* and *acetobacter*, as already mentioned, no such microorganisms were found. The two genera are most commonly associated with the suggested processes, but possibly not exclusively so. Their absence may be due to the concentrations of extracted DNA being practically ‘nil’.

Psimythion has for long been valued as an important white pigment in art and in cosmetics. In the period concerned here, it was also used as a mineral constituent of various medicines. Studying the material culture of the past on the basis of its use alone is only one way of looking at it and as such, it is usually limiting. It leaves unexplored other areas, ranging from aspects of its manufacture to its perceived value and symbolism (if any) within the cultural framework that generated it. In the case of psimythion, what is perhaps most intriguing is the implicit empirical understanding, from the part of psimythion manufacturers, not only of the range and dynamics of the chemical reactions, both biotic and abiotic, taking place within the pot, but also of their ability to control them. ‘Oxos’, its composition and properties, holds centre-stage and a better understanding of its role in early chemical synthesis of lead- and copper-based minerals is perhaps timely.
